# Selective Colorimetric Detection of Nitrite in Water using Chitosan Stabilized Gold Nanoparticles Decorated Reduced Graphene oxide

**DOI:** 10.1038/s41598-017-14584-6

**Published:** 2017-10-27

**Authors:** Baishnisha Amanulla, Selvakumar Palanisamy, Shen-Ming Chen, Te-Wei Chiu, Vijayalakshmi Velusamy, James M. Hall, Tse-Wei Chen, Sayee Kannan Ramaraj

**Affiliations:** 1 PG & Research department of Chemistry, Thiagarajar College, Madurai-09, Tamilnadu, India; 2 Electroanalysis and Bioelectrochemistry Lab, Department of Chemical Engineering and Biotechnology, National Taipei University of Technology, No. 1, Section 3, Chung-Hsiao East Road, Taipei 106, Taiwan, ROC; 3 Department of Materials and Mineral Resources Engineering, National Taipei University of Technology, 1, Sec. 3, Zhongxiao E. Rd., Taipei 106, Taiwan; 4 Division of Electrical and Electronic Engineering, School of Engineering, Manchester Metropolitan University, Manchester, M1 5GD United Kingdom

## Abstract

Excess nitrite (NO_2_^-^) concentrations in water supplies is considered detrimental to the environment and human health, and is associated with incidence of stomach cancer. In this work, the authors describe a nitrite detection system based on the synthesis of gold nanoparticles (AuNPs) on reduced graphene oxide (rGO) using an aqueous solution of chitosan and succinic acid. The AuNPs-rGO nanocomposite was confirmed by different physicochemical characterization methods including transmission electron microscopy, elemental analysis, X-ray diffraction, UV-visible (UV-vis) and Fourier transform infrared spectroscopy. The AuNPs-rGO nanocomposite was applicable to the sensitive and selective detection of NO_2_^−^ with increasing concentrations quantifiable by UV–vis spectroscopy and obvious to the naked eye. The color of the AuNPs-rGO nanocomposite changes from wine red to purple with the addition of different concertation of NO_2_^−^. Therefore, nitrite ion concentrations can be quantitatively detected using AuNPs-rGO sensor with UV-vis spectroscopy and estimated with the naked eye. The sensor is able to detect NO_2_^−^ in a linear response ranging from 1 to 20 μM with a detection limit of 0.1 μM by spectrophotometric method. The as-prepared AuNPs-rGO nanocomposite shows appropriate selectivity towards NO_2_^−^ in the presence of potentially interfering metal anions.

## Introduction

In recent years, metal nanoparticles have gained immense attention in various disciplines due to their unique physicochemical properties in terms of large surface area^1^, excellent adsorption characteristics^2^ and high electro-catalytic activity^3^. The unique properties of metal nanoparticles render them suitable to applications across a range of disciplines including catalysis^4^, chemical sensing^5^, biolabeling^6^ and photonics^7^. In particular, gold nanoparticles (AuNPs) have shown superior properties when compared to other metallic nanoparticles^8–10^. Due to their high molar extinction coefficient^11^, strong localized surface Plasmon resonance^12^, distance dependent optical properties^13^, stability^14^, and strong, well-defined color color change^15^, AuNPs have been widely applied for the selective probe for detection of anions^16,17^.

Among the three-inorganic nitrogen-containing nutrients (NH_4_^+^, NO_2_^−^, and NO_3_^−^), nitrite (NO_2_^−^) is essential nutrients for the growth of plants^18^. Nitrites (NO_2_^−^) are widely used for the preservation of food and curing of meat, with an acknowledged toxicity through the excessive uptake of NO_2_^−^^19^. In humans, high concentrations of NO_2_^−^ are associated with a number of medical issues such as methemoglobinemia, gastric cancer, and hypertension due to formation of carcinogenic N-nitroso compounds^19,20^. Moreover, NO_2_^−^ reacts with oxyhemoglobin in the blood and causes methemoglobinemia^18^. The World Health Organization set the fatal dose of NO_2_^−^ as 1.0 mg/L^21^. Given the reactivity of NO_2_^−^, a method for the rapid and accurate detection of NO_2_^−^ has great potential to help reduce the associated health risks. Analytical techniques developed for determination of NO_2_^−^ include spectrophotometry^22^, electrochemistry^23^, fluorescence^20^, chromatography^24^, chemiluminescence^25^ and surface-enhanced Raman scattering^26^. However, these methods have limited application in the routine detection of NO_2_^−^ due to a dependency on expensive reagents, instrumentation, long duration incubation periods, and highly skilled operators. Nonetheless, the sensitive, selective determination of NO_2_^−^ utilizing these technologies represents a significant challenge.

**Figure 1:**
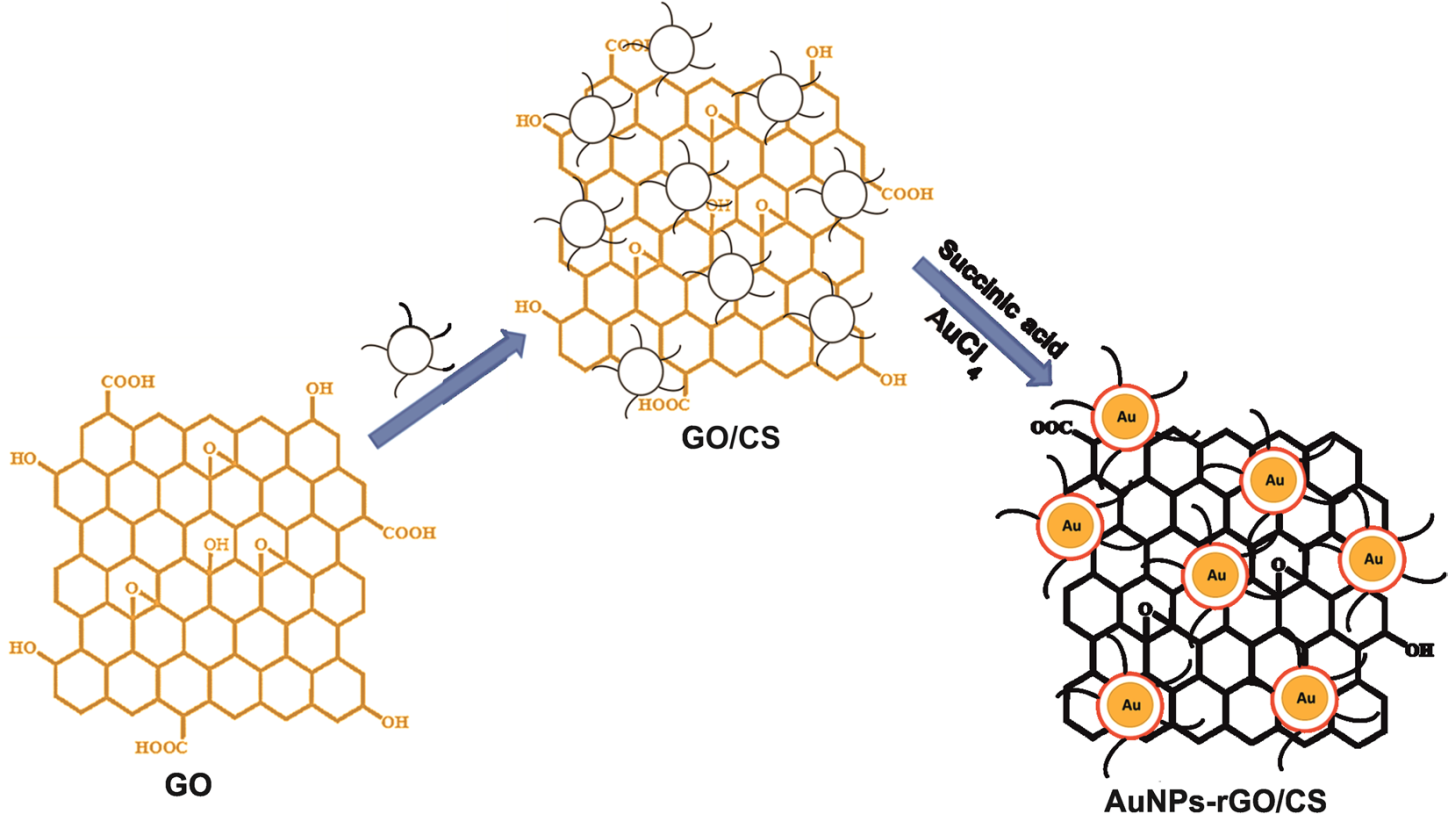
Schematic illustration for the synthesis of chitosan stabilized AuNPs-rGO composite. Abbreviations; GO - graphene oxide; CS - chitosan.

Due to its simplicity, high sensitivity, selectivity, easy operation, cost effectiveness and fast response^27^, colorimetric detection is widely applied to the trace level detection of NO_2_^−^. Recently, metal nanoparticles have been widely used for colorimetric detection of NO_2_^−^. As an alternative, reduced graphene oxide (rGO) possesses large specific surface area, and extraordinary mechanical, thermal, and electrical properties^28^. Owing to its unique properties, rGO has served as an excellent support for synthesis of nanoparticles decorated rGO or graphene composites. Furthermore, different chemical and electrochemical reduction methods have been used for the synthesis of metal nanoparticles decorated graphene composites including AuNPs-rGO composite. In addition, AuNPs decorated rGO in the presence of chitosan have also been well documented^29–31^. In the present work, we have synthesized chitosan stabilized AuNPs-rGO nanocomposite in presence of succinic acid for the first time (Fig. 1) and applied this to the colorimetric detection of NO_2_^−^. The selectivity and practicality of the NO_2_^−^ sensor has been critically studied and discussed.

## Results and Discussion

### Characterizations of AuNPs-rGO composite

The AuNPs-rGO composite were synthesized using chitosan and succinic acid as a reducing agent at 60 °C. Initially, GO was combined with chitosan to form a GO/chitosan composite. Succinic acid was utilized as a reducing and stabilizing agent for synthesis of the AuNPs-rGO composite. The reduction rate of AuCl_4_^−^ is enhanced by the presence of chitosan due the increase of the electrostatic interaction between NH_3_^+^ and AuCl_4_^−^ due to the higher degree of protonation in the amino groups of chitosan. A schematic representation for the synthesis of chitosan stabilized AuNPs/rGO composite is shown in Fig. 1.

**Figure 2:**
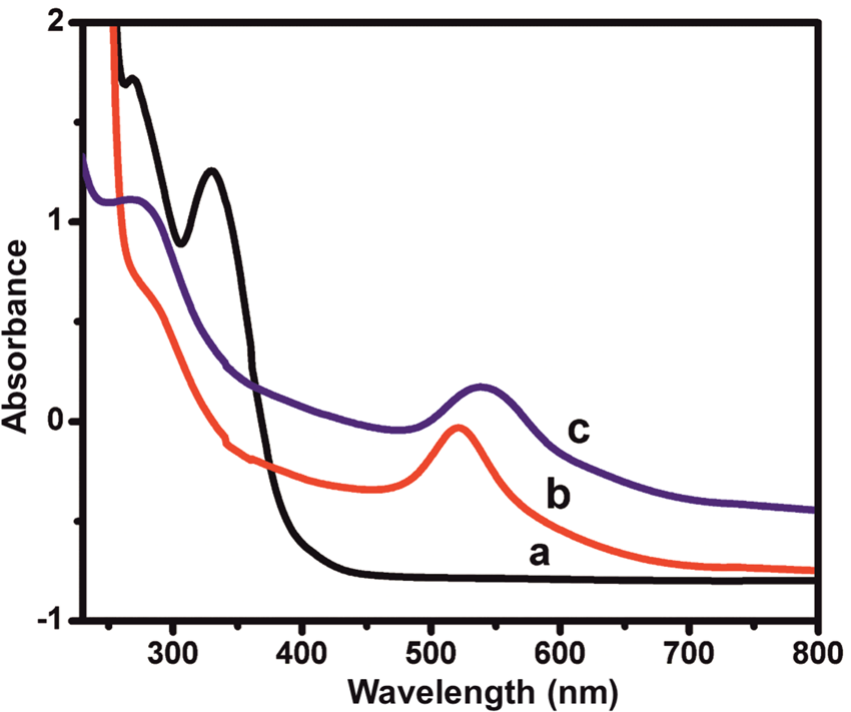
UV-vis spectra of the (**a**) GO, (**b**) AuNPs and (**c**) AuNPs^−^ rGO.

The formation of the AuNPs/rGO composite was confirmed by UV-vis spectroscopy. Figure 2 displays the UV-vis spectra of a) GO, b) AuNPs and c) AuNPs− rGO. UV-vis spectrum of GO shows 2 maximum bands at 249 and 344 nm, which are due to the π → π* transition of aromatic C–C bonds and n → π* transition of C = C^30^. UV-vis spectrum of AuNPs shows a sharp absorption maximum at 525 nm due to the surface Plasmon resonance of AuNPs^30^. UV-vis spectrum of AuNPs-rGO shows 2 maximum bands with red shifts at 536 and 266 nm, which are due to transition of aromatic C–C of rGO and surface Plasmon resonance of AuNPs^32^. The results confirm formation of the AuNPs-rGO composite.

**Figure 3:**
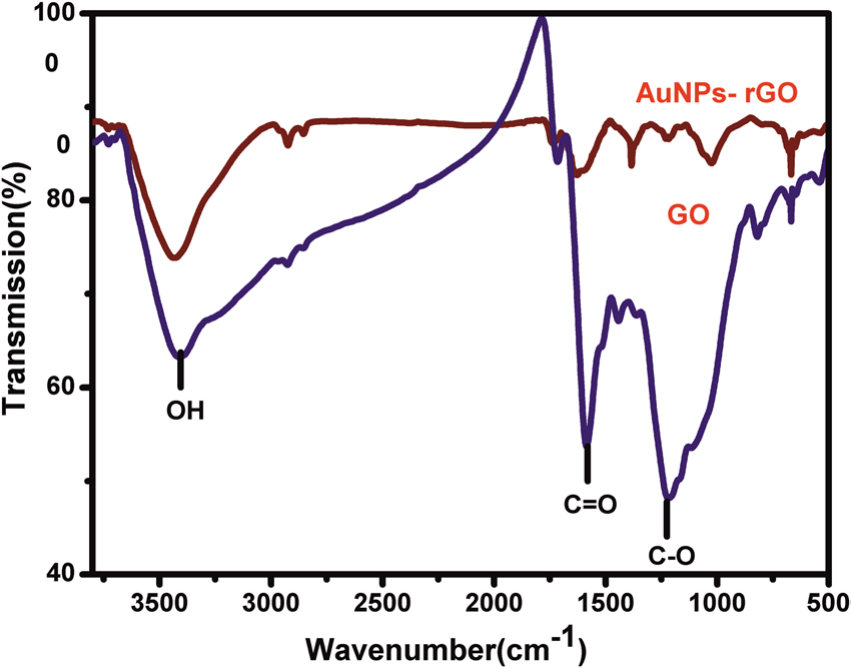
FTIR spectra of (**a**) GO and (**b**) AuNPs-rGO.

The formation of AuNPs-rGO composite was further confirmed by Fourier transform infrared spectroscopy (FTIR) and X-ray diffraction (XRD). Figure 3 shows the FTIR spectra of a) GO and b) AuNPs-rGO composite. The FTIR spectrum of GO shows the broad and intense absorption peaks at 3435, 1723 and 1245 cm^−1^ corresponding to the stretching vibrations of –OH, –C = O (carbonyl) and C-O (epoxy) groups, respectively^33^. Conversely, the characteristic absorption peaks of –OH, –C = O in the AuNPs-rGO composite decrease dramatically, indicating the successful transformation of GO to RGO^33^.

**Figure 4:**
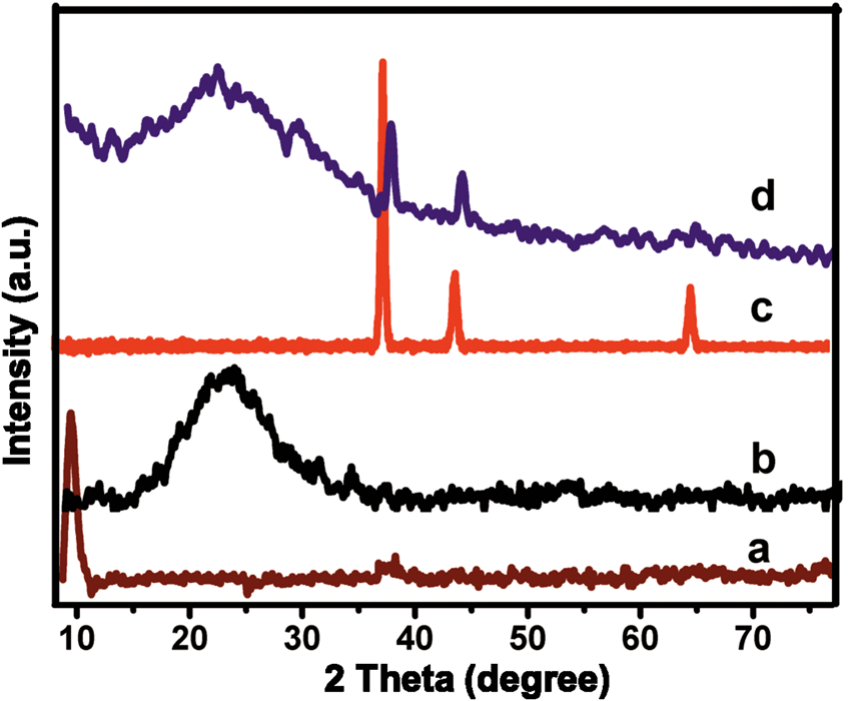
XRD profiles of (**a**) GO, (**b**) rGO, (**c**) AuNPs and (**d**) AuNPs-rGO.

Figure 4 shows the XRD profiles of a) GO, b) rGO, c) AuNPs and d) AuNPs-rGO composite. The XRD of GO showed a sharp diffraction peak at 2θ = 11.20, suggesting the complete exfoliation of graphite. XRD of rGO shows a sharp diffraction peak at 24.30 with the interlayer d-spacing of 0.45, which is due to the restoration of C-C (sp^2^) bonding in rGO^33^. The XRD of AuNPs-rGO composite shows four sharp diffraction peak at 38.20, 44.30, 65.20, 78.50, which are related to (111), (200), (220) and (311) planes of face centered cubic Au (JCPDS No. 04-0784). The result confirm that the Au(I) has been successfully reduced to Au(0)^32^. The average diameter of the AuNPs on AuNPs-rGO composite was calculated using the Scherer equation and indicates an average grain size of 25.6 ± 3 nm. The above results further support the formation of AuNPs-rGO composite.

**Figure 5:**
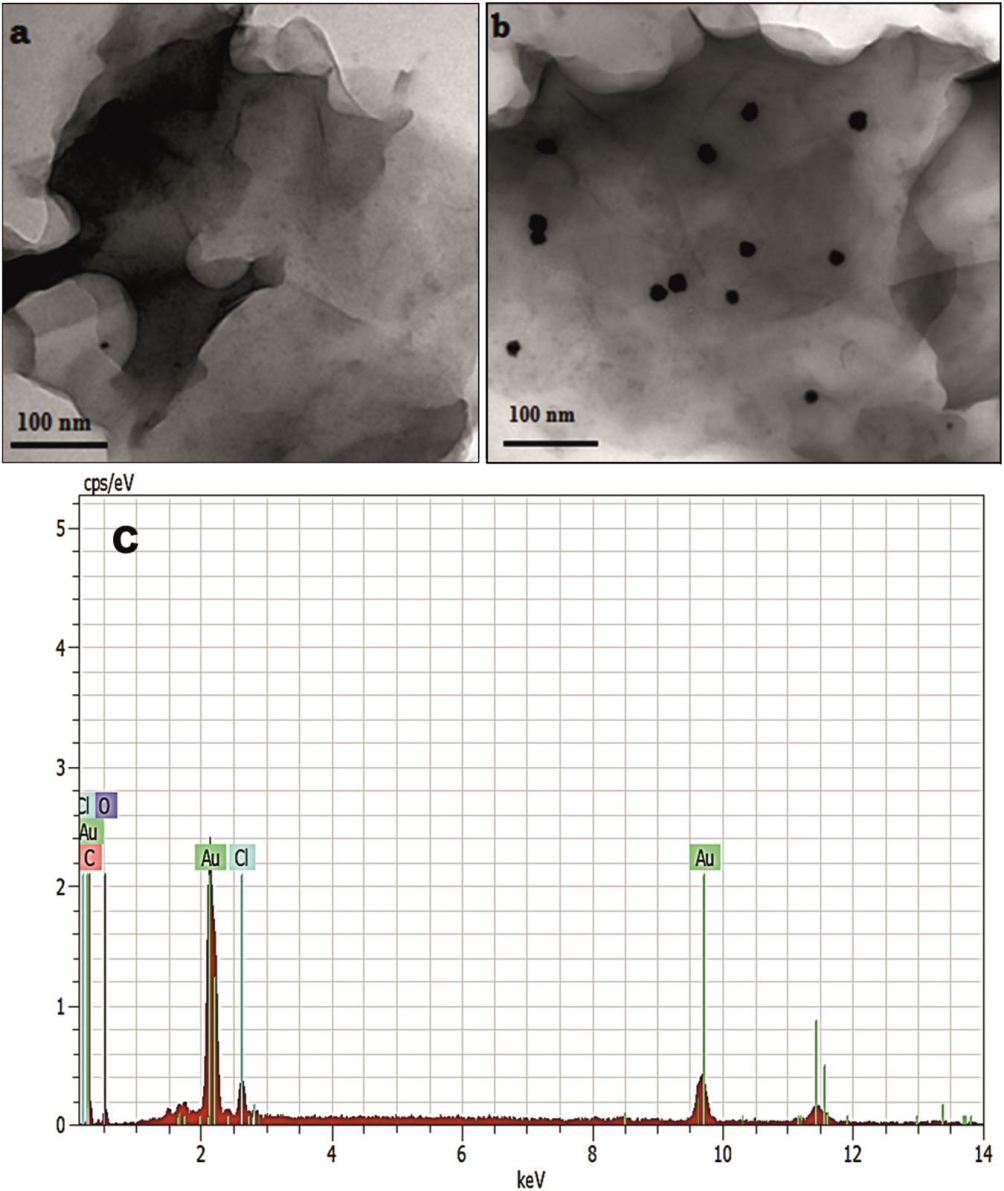
TEM images of (**a**) rGO, (**b**) AuNPs-rGO and corresponding EDS of AuNPs-rGO (**c**).

The surface morphologies of as-prepared AuNPs–rGO composite were investigated by transmission electron microscopy (TEM). Figure 5 displays the TEM images of a) rGO and b) AuNPs-rGO. It can be clearly seen that the spherical AuNPs are uniformly dispersed on the surface of rGO with an average diameter of 26 nm. Additionally, the TEM image of rGO shows the laminar structure with the association of few layers of nanosheets. The average diameter of AuNPs (26 nm) in AuNPs-rGO composite is in good agreement with the XRD results. As shown in Fig. 5c, the elemental analysis (EDS) confirms the presence of carbon, oxygen and metallic gold in AuNPs-rGO composite. The above results validate the formation of AuNPs-rGO composite.

**Figure 6:**
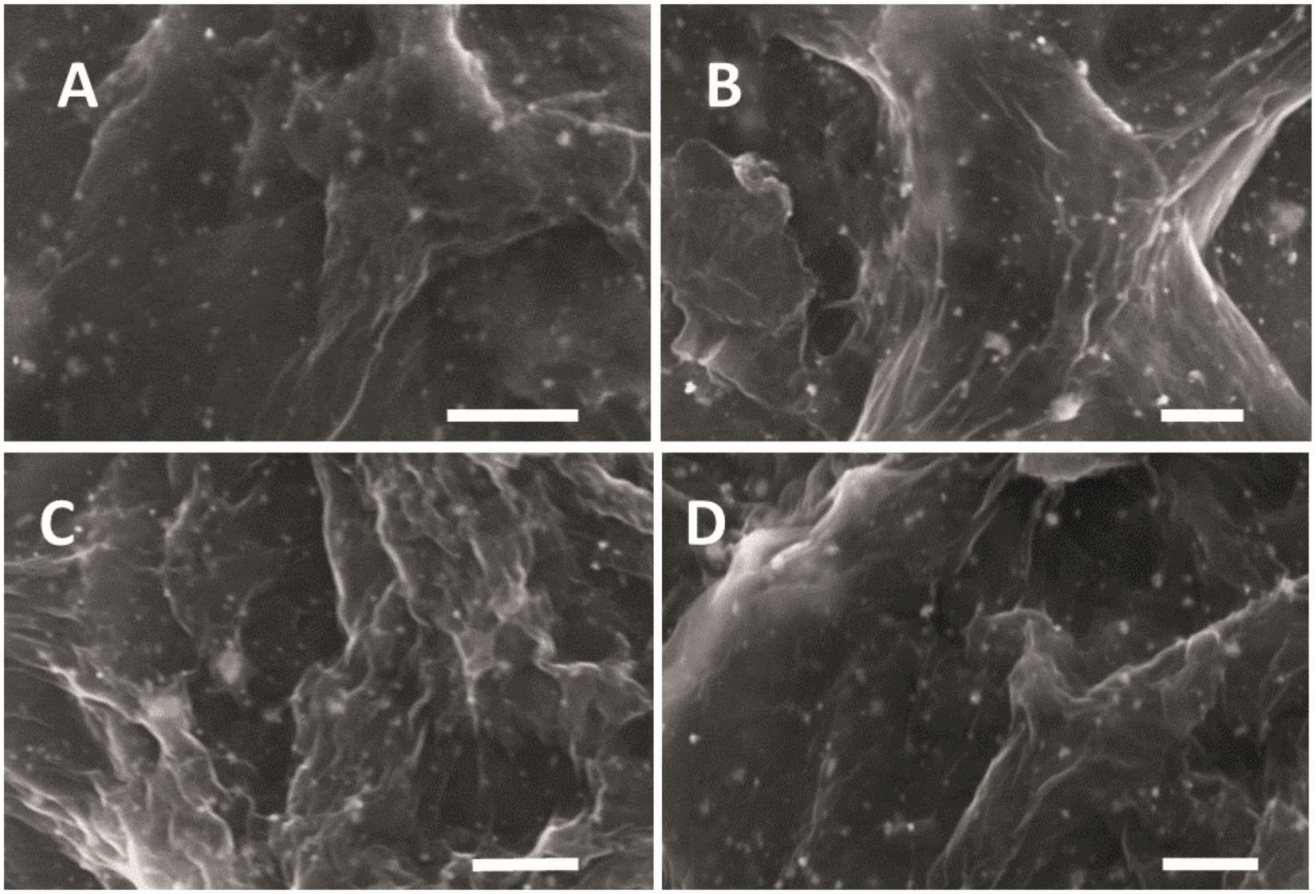
SEM images of AuNPs-rGO composite with 0.1 g (**A**), 0.3 g (**B**), 0.5 g (**C**) and 0.7 g (**D**) loading of chitosan. Scale bar = 500 nm.

To optimize the loading of chitosan, the AuNPs-rGO composite was prepared by the addition of 0.1 g (A), 0.3 g (B), 0.5 g (C) and 0.7 g (D) chitosan and corresponding scanning electron microscopic (SEM) images are shown in Fig. 6. The SEM images of AuNPs-rGO composite clearly reveals that 0.5 g addition of chitosan shows a more uniform morphology than the 0.1, 0.3, or 0.7 g. Hence, 0.5 g chitosan is considered the optimum for synthesis of AuNPs-rGO composite.

**Figure 7:**
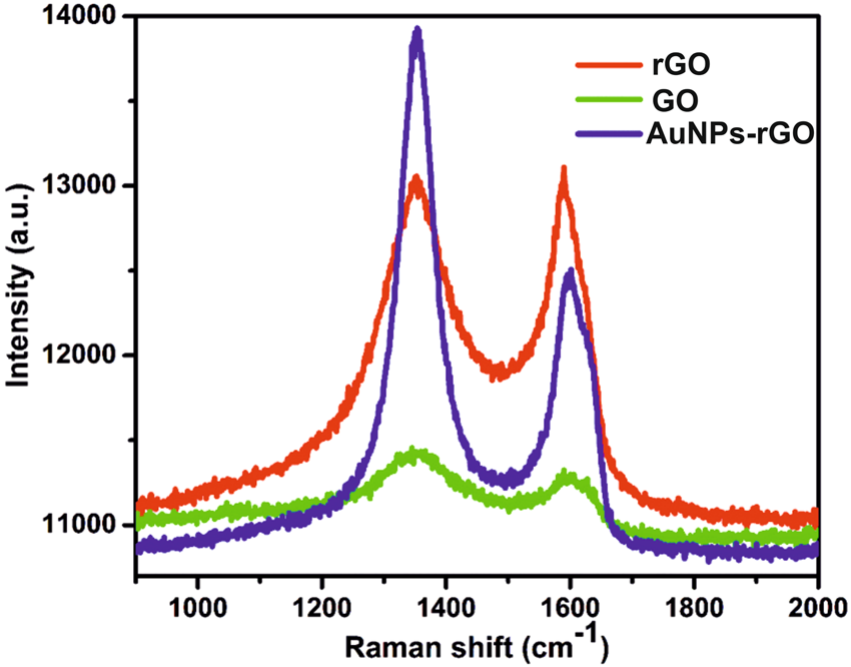
Raman spectra of rGO (red line), GO (green line) and AuNPs-rGO (blue line).

Raman spectroscopy is an ideal technique to confirm the transformation of GO to rGO and the representative Raman spectra of rGO (red line), GO (green line) and AuNPs-rGO (blue line) are shown in Fig. 7. The Raman spectrum of GO (green color) shows distinct D and G peaks at 1344 and 1583 cm^−1^, which are attributed to the vibrations of sp^3^ and sp^2^ carbon atom domains of graphite. The intensity ratio of *I*_D_/*I*_G_ was higher in rGO (1.02) and rGO-AuNPs (1.05) than GO (0.92), which clearly shows the transformation of GO to rGO.

### Colorimetric determination of NO_2_^−^

**Figure 8:**
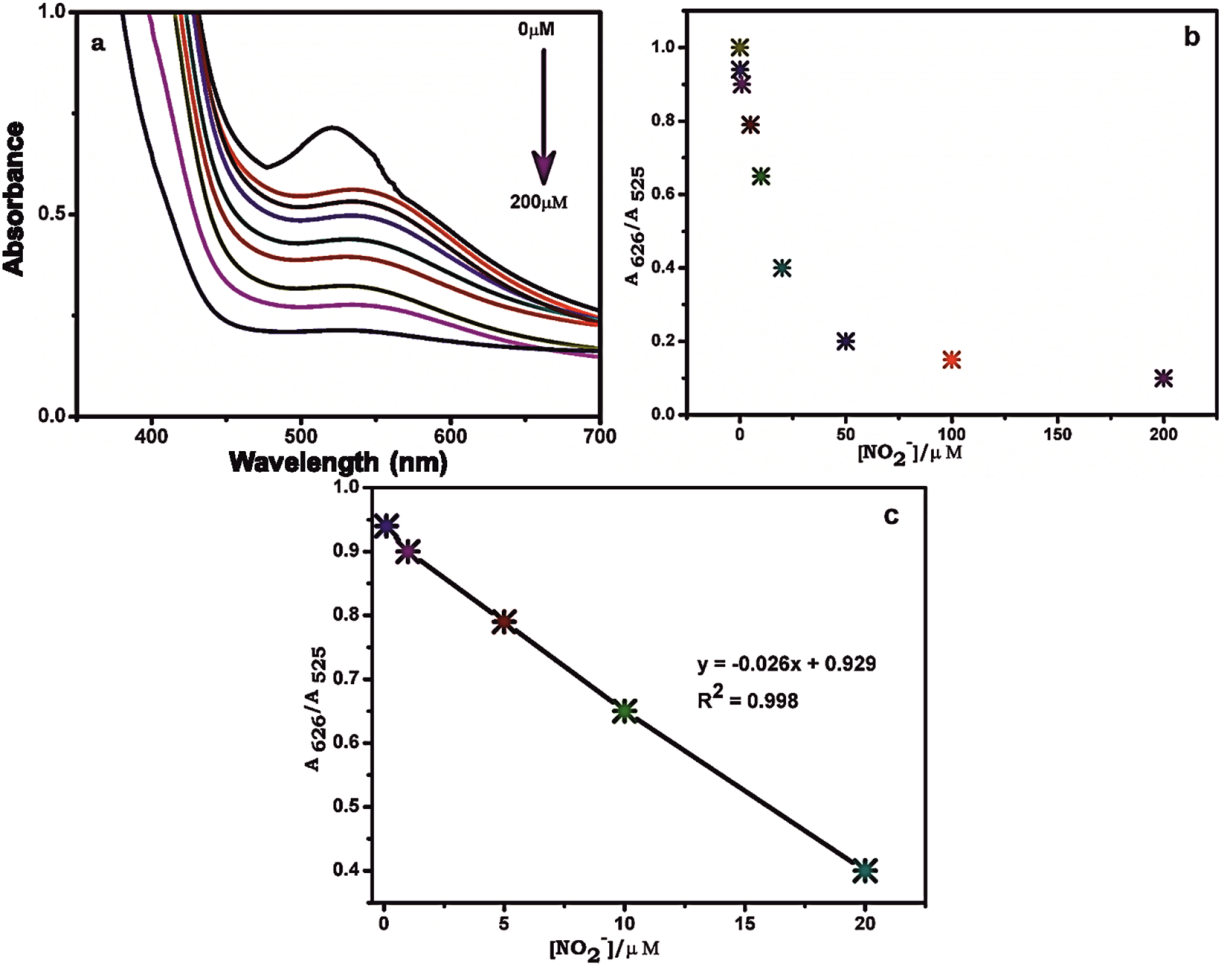
(**a**) UV-vis absorption spectra of AuNPs-rGO for i) 0 μM, ii) 0.1 μM, iii) 1 μM, iv) 5 μM, v) 10 μM, vi) 20 μM, vii) 50 μM, viii) 100 μM and ix) 200 μM additions of NO_2_^−^. (**b**) The plot of absorbance ratio (A626/ A525) vs. [NO_2_^−^]. (**c**) Linear plot of A626/A525 vs. [NO_2_^−^] from 0.1 to 20 μM.

To demonstrate the capacity detect and quantify low levels of NO_2_^−^, UV-vis spectroscopy was used to determine the concentration of NO_2_^−^ in an aqueous solution. Figure 8a shows the UV-vis absorption spectra of AuNPs-rGO for the addition of different concentration of NO_2_^−^ (0–200 µM) into the AuNPs-rGO composite solution. As can be seen, the peak at 525 nm was shifted and a new peak appeared at 626 nm upon addition of NO_2_^−^. An increase of NO_2_^−^ concentration from 0.1 µM to 200 µM resulted in an increase of absorbance in the 626 nm region increased and a corresponding decrease in absorbance in the 525 nm region (Fig. 8b). The value of A685/A520 increased linearly with a lowest detection capability of 0.1 µM. The calibration curve for the value of A685/A520 vs. [NO_2_^−^] was linear in the detection range from 1 to 20 µM with a correlation coefficient (R^2^) of 0.998 (Fig. 8c). The lowest detection level of our sensor is well below for the maximum level of NO_2_^−^ in drinking water (21.7 µM) as set by the Environmental protection agency (EPA). It was noted that the results confirmed the proposed material as highly suitable to real-time, on-site detection of NO_2_^−^ in drinking water. It was also noted that the analytical performance of AuNPs-rGO nanocomposite is superior towards NO_2_^−^ than AuNPs prepared in the absence of rGO (data not included). The obtained analytical results of our sensor were compared with the previously reported sensors, and the analytical results gained (LOD and linear response range) are comparable with reported nitrite sensors including electrochemical systems^19,20,23,27,34–36^. It also noted that the analytical performance of sensors is more comparable to previously reported colorimetric detection of NO_2_^−^ based on gold nanomaterials (AuNPs and nanorods)^37–40^. Accordingly, the present sensor demonstrates practical potential for the sensitive detection of nitrite at low levels.

### Selective detection and mechanism of NO_2_^−^

**Figure 9:**
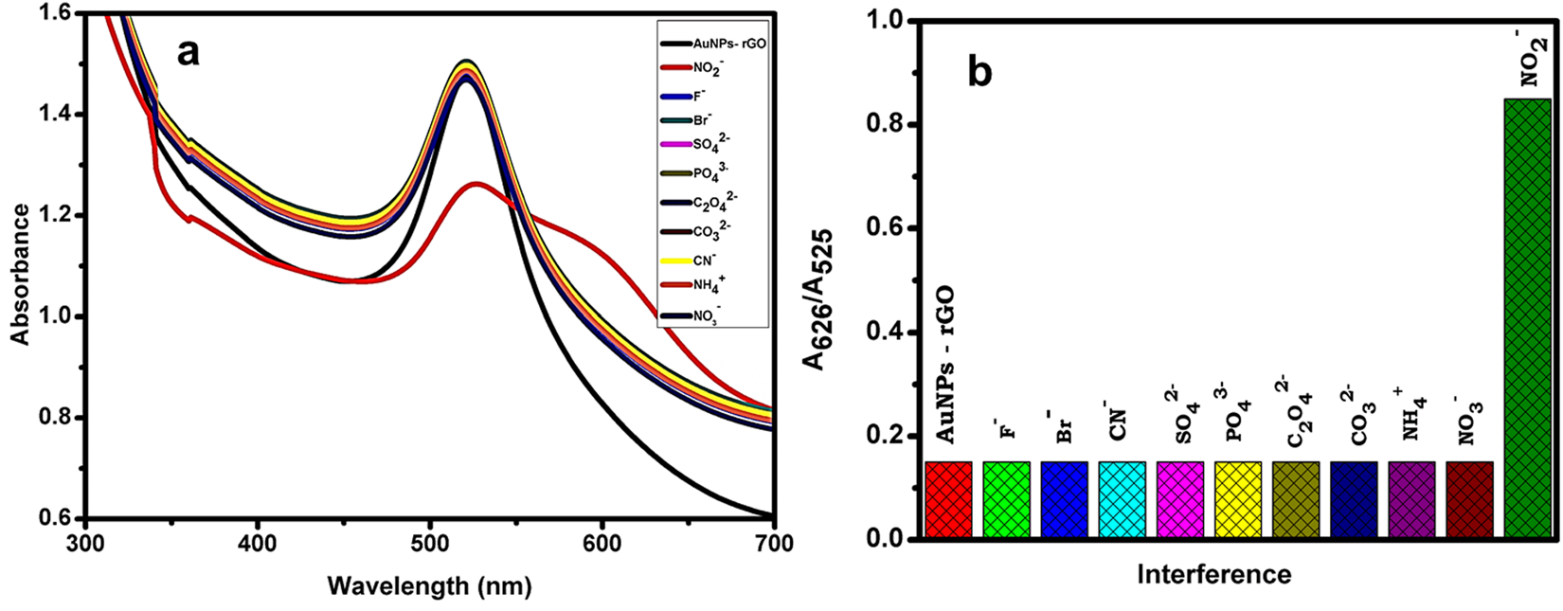
(**a**) UV-vis absorption spectra of AuNPs-rGO with different anions. (**b**) The corresponding absorbance ratio (A626/ A525) in the presence of various anions.

The selective recognition ability of NO_2_^−^ on AuNPs-rGO composite was examined in the presence of different metal anions. The apparent color changes were measured by UV-vis spectra and the corresponding results are shown in Fig. 9a and b. The observed results clearly show that 20 µM addition of F^−^, Br^−^, CN^−^, SO_4_^2−^, PO_4_^3−^, C_2_O_4_^2−^ and CO_3_^2−^ does not show any significant change in the UV-vis spectra of the composite, while the addition of 5 µM NO_2_^−^, the absorbance peak of AuNPs at 525 nm dramatically decreased and a new peak was observed at 626 nm. The excellent selectivity of the sensor towards NO_2_^−^ attributed to the high specificity of the nitrous acid with amines of chitosan in the AuNPs-rGO composite.

**Figure 10:**
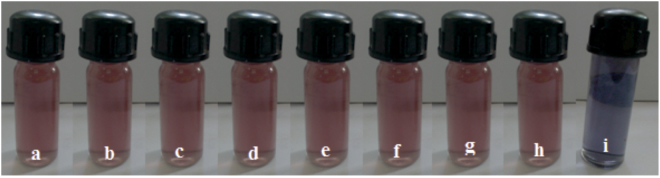
Digital photograph images of AuNPs− rGO composite with the addition of 20 µM of different anions; (**a**) AuNPs-rGO, (**b**) F^−^, (**c**) Br^−^, (**d**) CN^−^, (**e**) SO_4_^2−^, (**f**) PO_4_^3−^, (**g**) C_2_O_4_^2−^, (**h**) CO_3_^2−^ and 5 µM addition of (**i**) NO_2_^−^.

Under the optimized conditions, the color change of the composite in the presence of different ions was measured and recorded using a digital camera. As shown in Fig. 10, the AuNPs-rGO composite shows red color in the absence of metal anions, and the color was not affected by the addition of 20 µM F^−^, Br^−^, CN^−^, SO_4_^2−^, PO_4_^3−^, C_2_O_4_^2−^, CO_3_^2−^, NH_4_^+^ and NO_3_^−^. However, the color changes from red to purple upon addition of 5 µM of NO_2_^−^ to the composite solution. The color change from wine red to purple is due to the aggregation of chitosan stabilized AuNPs-rGO, arising from a closer formation of the metal nanoparticles. The presence of chitosan enhances the stability of AuNPs-rGO in aqueous solutions. The results demonstrate that the proposed method can be used for the selective direct detection of NO_2_^−^ both with the naked eye, and with spectrophotometric methods.

### Determination of NO_2_^−^ in water samples

To evaluate the practical ability of the sensor, the concentration of NO_2_^−^ was determined for a range of collected water samples by UV-vis spectrophotometry. Three different tap water samples were collected from the Thiagarajar college campus, Madurai and all obtained water samples were shown to be NO_2_^−^ free. A known quantity of NO_2_^−^ (5 µM) was added and the water samples were re-analyzed. The concentration of NO_2_^−^ in the spiked water samples was calculated from the linear plot of A626/A525 as shown in Fig. 8c, and the obtained recoveries tabulated in Table 1. The average recovery of NO_2_^−^ in the adulterated water samples was determined as 97.0% with a relative standard deviations (RSD) of 5.0% (n = 5). The results demonstrate the potential application of the proposed AuNPs-rGO sensor in the detection of NO_2_^−^ in water and food samples.

**Table 1:** Determination of NO_2_^−^ in water samples using AuNPs-rGO composite by UV-vis spectrophotometry. RSD is relative to five measurements.

Sample	Detected (µM)	Added (µM)	Found (µM)	Recovery (%)	RSD (%)
Water sample 1	Not detectable	5.0	4.92	98.4	4.7
Water sample 2		5.0	4.89	97.8	4.9
Water sample 3		5.0	4.86	97.2	5.2
Water sample 4		5.0	4.76	95.2	5.1
Water sample 5		5.0	4.79	95.8	4.4
Water sample 6		5.0	4.82	96.4	4.7
Water sample 7		5.0	4.91	98.2	6.0

The stability and reproducibility of the sensor was also evaluated under the experimental conditions outlined in Fig. 8a. The AuNPs-rGO/CS composite shows appropriate stability (~98.2%) through continuous monitoring over a period of 14 days (figure not shown). In addition, the fabricated sensor shows appropriate reproducibility with an RSD of 5.3% across five independent sensors in the detection of 5 µM NO_2_^−^ (figure not presented). Accordingly, the fabricated AuNPs-rGO/CS composite is suitable for long duration and accurate detection of NO_2_^−^.

## Conclusions

In conclusion, a novel and selective colorimetric sensor has been developed for determination of NO_2_^−^ using AuNPs-rGO composite as a colorimetric probe. The as-prepared nanocomposite has been thoroughly characterized and the obtained results confirmed the formation of AuNPs-rGO composite. Under optimized conditions, the lowest detection level of our sensor is 0.1 µM and is well below for the maximum level of NO_2_^−^ in drinking water (21.7 µM) that set by the Environmental protection agency (EPA). The sensor also showed high specificity towards NO_2_^−^ in the presence of range of metal anions. The sensor showed acceptable recovery towards NO_2_^−^ in water samples, which authenticates its potential real time sensing ability. We believe that as-prepared chitosan stabilized AuNPs-rGO composite represents a simple, robust, inexpensive material with great potential for application in the sensitive and low-level detection of NO_2_^−^.

## Experimental

### Materials and Methods

Fine graphite powder (<50 μm) was received from Sigma-Aldrich, India. Tetrachloroauric (III) acid trihydrate, chitosan, sulfuric acid (AR grade), potassium permanganate, hydrogen peroxide (30%), sodium nitrate, sodium nitrite, sodium fluoride, sodium bromide, sodium thiocyanate, calcium oxalate, disodium phosphate, sodium bicarbonate, sodium sulphate, and succinic acid were obtained from Merck, India. All chemicals were of analytical grade and used as received. The stock solutions were prepared using doubly distilled water and the experiments were performed under ambient conditions.

UV–vis spectral measurements were performed using a Jasco (V-560) spectrometer. The morphological studies of the as-synthesized composite were characterized by FEI Tecnai G2 20 S-TWIN TEM with an accelerating voltage of 200 kV. FEI Tecnai G2 20 S-TWIN TEM attached BRUKER AXS elemental analyzer was used for the EDS and elemental mapping of the composite. XRD analysis was performed using from Panalytical X′ per PRO X-ray diffractometer equipped with Cu Kα radiation (λ = 0.15406 nm). FTIR was performed by a Shimadzu model FT-IR spectrometer.

### Synthesis of GO

GO was synthesized from natural graphite based on Hummers method^30^, with some modification. Briefly, 2 g of graphite powder was added into the mixture of 1 g of NaNO_3_ and 50 mL H_2_SO_4_ and stirred for 30 min at 0 °C. Subsequently, about 12 g of KMnO_4_ was slowly added into the above mixture with continued stirring for 2 h. The temperature of the mixture was then heated to 35 ± 5 °C for 30 min. Approximately 150 mL of water was slowly added to the mixture and the solution was heated to 90 °C with vigorous stirring for 15 min. Then, 120 mL of H_2_O_2_ aqueous solution was added to the suspension until its color was changed to brilliant yellow. The obtained graphite oxide was washed three times with diluted HCl (5%) and doubly distilled water, and dried in a vacuum oven at 50 °C for 24 h.

### Synthesis of AuNPs-rGO composite

To synthesize AuNPs-rGO composite, 0.5 g of chitosan was added to the 50 mL of GO suspension (1 mg mL^−1^) and the mixture was stirred for 30 min. Simultaneously, 0.1 M of succinic acid solution and 50 mL of HAuCl_4_ (1 mM) solution was added. The mixture was heated to 60 °C with reflux under magnetic stirring until the color turned to wine red. The rGO and AuNPs were also prepared using similar method without AuNPs and GO. The as-synthesized AuNPs-rGO composite was dried in an air oven.

### Colorimetric detection of NO_2_^−^

The colorimetric selective detection of NO_2_^−^ was carried out at the room temperature in a natural pH. First, 100 µL of NO_2_^−^ solution with different concentrations were added to 900 µL of AuNPs-rGO composite (0.5 mg mL^−1^). The change of color of the composite was observed by the naked eye or UV-vis spectroscopy. The selectivity of sensor was determined by the same method^41,42^ in the presence of metal ions including F^−^, Br^−^, CN^−^, SO_4_^2−^, PO_4_^3−^, C_2_O_4_^2−^ and CO_3_^2−^.
